# Green roofs and pollinators, useful green spots for some wild bee species (Hymenoptera: Anthophila), but not so much for hoverflies (Diptera: Syrphidae)

**DOI:** 10.1038/s41598-023-28698-7

**Published:** 2023-01-26

**Authors:** Jeffrey Jacobs, Natalie Beenaerts, Tom Artois

**Affiliations:** UHasselt, Centre for Environmental Sciences, Agoralaan Gebouw-D, 3590 Diepenbeek, Belgium

**Keywords:** Biodiversity, Urban ecology, Ecosystem services

## Abstract

Urbanisation has become one of the major anthropogenic drivers behind insect decline in abundance, biomass and species richness over the past decades. As a result, bees and other pollinators' natural habitats are reduced and degraded. Green roofs are frequently recommended as ways to counter the negative impacts of urbanisation on nature and enhance the amount of green space in cities. In this study we evaluated the pollinator (more specifically wild bees and hoverflies) diversity, abundance and species richness on twenty green roofs in Antwerp, Belgium. We analysed the influence of roof characteristics (age, surface area, height, percent cover of green space surrounding each site) on species richness or abundance of pollinators. In total we found 40 different wild bee species on the green roofs. None of the physical roof characteristics appear to explain differences in wild bees species richness and abundance. Neither could we attribute the difference in roof vegetation cover, i.e. roofs build-up with only *Sedum* species and roofs with a combined cover of *Sedum*, herbs and grasses, to differences in diversity, abundance, or species richness. We found a positive trend, although not significant, in community weighted mean body size for wild bees with an increase in green roof surface area. Roof wild bee communities were identified as social polylectic individuals, with a preference for ground nesting. Only eleven individuals from eight different hoverfly species were found. Our results show that green roofs can be a suitable habitat for wild bee species living in urban areas regardless of the roofs’ characteristics, but hoverflies have more difficulties conquering these urban green spaces.

## Introduction

Urbanisation—the gradual shift in residence of the human population from rural to urban areas—combined with the overall growth of the human population causes an increase in habitat loss, more fragmentation and an overall change in habitat quality^1^. One of the major effects of this urbanisation trend is a serious threat to biodiversity on a global scale^2,3^. Increased city area results in species habitat loss, increased spatial distance between remaining pockets of green, and an overall change in habitat quality^1^. These factors have caused an overall decline in insect abundance, biomass and species richness^4,5^. Whether the rates of decline for insects are on par with or exceed those for other groups remains unknown^6^.

Pollinators are no exception, and together with other functional insect groups also suffer from stressors such as parasites, pesticides and a lack of flowers^7^. Pollination is vitally important to ecosystems and crop production, with a staggering 87% (~ 310.000 species) of all flowering plants depending on animal pollination^8^. An annual market value of $235–577 billion worldwide, is directly attributable to animal pollination^9^. In the north-temperate zone (e.g. Europe) bees, hoverflies and lepidopterans dominate pollination, whereas, in other parts of the world other pollinators, such as wasps and beetles, may be just as important^8^. Domesticated honeybees are often used in agricultural areas, although wild bees are the more efficient pollinators^10^. In addition, the pollination service's long-term stability is dependent on bee species richness and abundance^11,12^. Global honeybee stocks have increased in the past fifty years, while wild bees appear to have declined substantially over this period^7,13^. The decline of pollinator species and their distribution is strongly influenced by habitat loss and fragmentation and is further magnified by global warming^9^. Although it is clear that urbanisation has an effect on pollinator abundance and species richness, the effects can go both ways^14,15^. Overall, lower pollinator species richness and abundance with a relative increase in the proportion of generalist pollinators, such as bumblebees^16^ are associated with an increase in urbanisation^17,18^. This is not necessarily a positive evolution, since specialist species appear to have superior pollinator effectiveness compared to generalist species^19^, at least for some plant species.

To counter the negative environmental effects of urbanisation and increase the total amount of green spaces in cities, the construction of green roofs is often promoted^20^. Green roofs can deliver several ecosystem services, such as benefits for water runoff and evapotranspiration^21^. They are also an efficient solution to mitigate the heat island effect^22^ or increase habitat connectivity for mobile arthropod species by acting as stepping-stones between habitats^23^. In addition, green roofs can be suitable habitats for a wide variety of pollinator species^24–28^. These roofs produce pollen and nectar throughout the summer and include a variety of nesting locations^25,29^. Furthermore, the increased insulation and hence warmer microclimate at roof level create favourable habitat conditions for some pollinator species^30^. However, plant–pollinator networks in urban environments have fewer plant–insect interactions than those in semi-natural habitats^31^.

Urban settings have neutral or even positive effects on the biodiversity of several insect pollinator groups, particularly wild bee species^32^. Possibly because they are more sensitive to agricultural pesticides than other groups^33^. Whether common species of wild bees such as *Bombus terrestris* (Linnaeus, 1758) are more or less abundant than some decades ago remains unclear. There are maps of past and present distributions of bees in well studied countries such as the United Kingdom, but little information is available on how populations have evolved over time in terms of abundance^7^. Wild bees play a vital role in urban ecosystems as pollinators in gardens, parks, and other green spaces^14,30,34^, they respond to land-use change in a variety of ways, both within and between taxa^35,36^. Some habitats within cities support a high diversity of flowering plants and pollinating insects, although the shift to highly generalized habitats with less variation such as green roofs, may make pollination services in urban areas more susceptible to future disturbance events^37^. Extensive green roofs, which consist of a 5–20 cm deep layer of homogeneous, shallow, rocky substrate, are the most prevalent type of green roof in Europe. Allochthonous plants, such as species of *Sedum* or other drought-tolerant plants, are commonly used since they require little maintenance and are resistant to summer drought^38^. Plant and associated animal communities on green roofs are expected to become more diverse over time, as natural systems go through successive transitions marked by increasing diversity^39^. Arthropods and microorganisms start colonizing green roofs immediately, as they are typically carried in with planting material or substrate^40,41^.

Wild bees use green roofs as a habitat on a regular basis^24,25,28^. There is, as expected, a positive impact on pollinator diversity and abundance on green roofs with an increasing diversity of entomophilous plant species^25,28,29,42,43^. Pollinator communities on green roofs are influenced by different green roof characteristics such as size^42,44,45^, height^23,27^, vegetation cover^28,46^ and the proportion of green space in the surrounding landscape^25^.The age of a green roof does not affect bee communities significantly^28^, although it affects abundance of other groups, such as Lepidoptera^43^, Collembola^47^ and spontaneous vascular plant richness^48^ in a positive way. Although correlations have been shown, the effects of urbanisation and green roof characteristics on pollinator communities are probably case-specific and differ between cities and climates.

Hoverflies are gaining popularity as beneficial species and alternative managed pollinators due to their significant involvement in pollination and other ecological services. In Europe more than 70% of animal-pollinated wildflower species are visited by hoverflies^49^. However, hoverflies visiting green roofs have received far less attention than wild bees. They depend on the availability of appropriate plant taxa such as Asteraceae and Crassulaceae for nectar and pollen^50^, and unlike bees they use the nectar as a source for ovarian development^51^. Hoverflies do not have a fixed home range and can transport pollen over greater distances than bees during foraging^52^. Moreover, during their larval development hoverflies are very restricted to specific microhabitats because of their diet^53,54^. Due to their specific ecology hoverflies need a variety in landscape characteristics. Hoverflies require both suitable habitats for their larvae and flower resources at landscape scales; however, due to dispersal, habitat fragmentation, and barriers in built structures (e.g. large buildings), such resource complementarity is less common in urban areas^55^. Previous studies have shown that the small number of ideal habitats in urban areas is probably the main cause of the higher sensitivity of hoverflies to urbanisation compared to bees^55,56^. Furthermore, size and morphology can have an impact on pollinator efficiency and determine the amount of pollen the hoverfly can carry^54^. Individuals' spatial scale at which they perceive their world is influenced by their dispersal capabilities. Hoverfly species can disperse anywhere from a few meters to 2 kms every day^57,58^, but most species only disperse over very short distances, except during mass migration events.

The aim of our study was to investigate whether roof characteristics influenced pollinator diversity, abundance and species composition of green roofs in an urban environment. Therefore, we investigated 20 large green roofs in the city of Antwerp, Belgium. More specifically we expected that (i) green roofs with a large surface area would have a higher pollinator richness, abundance and diversity. We analysed (ii) if other roof characteristics (e.g. height) influenced species richness, abundance or diversity of pollinators present. Furthermore, we hypothesised (iii) that green roofs with a greater floral richness, i.e. here having a mixed vegetation cover of *Sedum*, grass and herbs, have a higher diversity and abundance of pollinators than the *Sedum* monoculture roofs. Finally, we analysed community weighted means to determine the average community traits (body size, social behaviour, flower visiting and nesting type) of wild bee species on the green roofs. We compared these CWM results for the two main roof types (Sedum vs Sedum/herbs/grasses), as species use traits to maximize their fitness in a different environment.

## Materials and methods

### Study sites

Our study areas comprised 20 green roofs in the city of Antwerp, Belgium. The city of Antwerp (51° 13′ N, 4° 24′ E) comprises a total area of 204.5 km^2^ with ± 526.000 citizens (2413.1 inhabitants/km^2^). We sampled macro-invertebrates from the months of March until September in the years 2020 and 2021.

The average temperature during the sampling period of 2020 was 15.3 °C (± 0.1) and the average precipitation was 53.6 mm^59^. From August 5–16 2020 a heatwave occurred (i.e. the temperature was at least 25 degrees on five consecutive days or more, with at least 30 degrees being reached for three days;^59^). The average temperature during the sampling period of 2021 was 14.7 °C (± 0.1) and the average precipitation was 91.6 mm. During the months of June (121 mm), July (166.5 mm) and August (123 mm) 2021 the precipitation exceeded the overall averages of these months (June: 70.8 mm, July: 76.9 mm and August: 86.5 mm)^59^.

The percentage of grassland in the surrounding landscape within a radius of 300 m centred at the middle of each green roof was calculated using the software Google Earth Pro (version 7.3.6.9345) (see appendix table A7 and figure A6 in supplementary information).

### Green roof characteristics

On average the green roof surface was 280.6 m^2^ (range 8 m^2^–896 m^2^), the average age was 8.4y (range 3–14y), and their average height was 10.4 m (range 4–23 m; Table 1). The roofs are made up of a 5–20 cm layer of homogeneous, shallow rocky substrate. They are typically planted with species of *Sedum* or other drought tolerant plants (e.g. species of mosses and grasses such as *Calamagrostis epigejos* (Roth, 1788)). Roofs were separated into two groups according to the vegetation type *Sedum* roofs (Mid 1, Mid 2, Onyx, Eco 1, PM, RSL, RPBer, RPDeu, Iglo, Bell, Arena) and *Sedum*, herbs and grass roofs (Dis, Atlas, Eco 2, Ell, Hard, Bra, RPWil, Boek 1, Boek 2). We conducted two vegetation surveys on all roofs in June 2020 and 2021 (see appendix table A3 in supplementary information for an overview of the flora species per green roof).

**Table 1:** Overview of the roofs with their reference name, age, surface area, height above ground level and dominant vegetation.

Name roof	Reference	Location	Age (y)	Surface (m^2^)	Height (m)	Dominant vegetation
Middelheim 1	Mid 1	Antwerpen (N51.184°, E4.420°)	7	260	10	*Sedum*
Middelheim 2	Mid 2	Antwerpen (N51.184°, E4.419°)	7	45	10	*Sedum*
Onyx	Onyx	Berchem (N51.193°, E4.417°)	7	708	23	*Sedum*
District	Dis	Wilrijk (N51.169°, E4.394°)	13	320	9	*Sedum, herbs and grasses*
Atlas	Atlas	Antwerpen (N51.130°, E4.253°)	8	320	8	*Sedum, herbs and grasses*
Ecohuis 1	Eco 1	Borgerhout (N51.125°, E4.260°)	3	35	12	*Sedum*
Ecohuis 2	Eco 2	Borgerhout (N51.125°, E4.260°)	3	8	12	*Sedum, herbs and grasses*
Plantin Moretus museum	PM	Antwerpen (N51.218°, E4.398°)	5	84	15	*Sedum*
Ellerman	Ell	Antwerpen (N51.230°, E4.415°)	6	312	9	*Sedum, herbs and grasses*
Hardenvoort	Hard	Antwerpen (N51.135°, E4.251°)	5	630	20	*Sedum, herbs and grasses*
Red Star Line museum	RSL	Antwerpen (N51.135°, E4.241°)	10	408	10	*Sedum*
Brandweer	Bra	Antwerpen (N51.251°, E4.418°)	12	777	17	*Sedum, herbs and grasses*
Recycling park Wilrijk	RPWil	Wilrijk (N51.160°, E4.390°)	7	164	4	*Sedum, herbs and grasses*
Recycling park Berchem	RPBer	Berchem (N51.194°, E4.439°)	7	154	4	*Sedum*
Recycling park Deurne	RPDeu	Deurne (N51.237°, E4.457°)	7	142	4	*Sedum*
Urban childcare centre Strandloper	Iglo	Antwerpen (N51.225°, E4.380°)	9	896	5	*Sedum*
Administrative centre den Bell	Bell	Antwerpen (N51.205°, E4.399°)	12	85	21	*Sedum*
Boekenberg park 1	Boek 1	Deurne (N51.197°, E4.463°)	14	108	5	*Sedum, herbs and grasses*
Boekenberg park 2	Boek 2	Deurne (N51.197°, E4.462)	14	85	4	*Sedum, herbs and grasses*
OCMW Arena	Arena	Deurne (N51.199°, E4.459°)	13	72	6	*Sedum*

### Data collection

We sampled the green roofs every three weeks from March till September 2020 and 2021 to assess flying invertebrate biodiversity with randomly installed pan traps (diameter = 12 cm, height = 4 cm) of four different colours (blue, yellow, red and white;^60^). The pan traps were filled with clear propylene glycol and were emptied after 24 h. Invertebrates were stored in 70% ethanol. We also sampled the green roofs with the use of pitfall traps (diameter = 8 cm, height = 6 cm) to assess ground dwelling macro-invertebrate biodiversity^61,62^. We installed four pitfall traps at each site at random. The pitfall traps were covered with a lid to protect the trap from flooding with rain, and again propylene glycol was used to fill the traps to capture the invertebrates^63^. Every three weeks traps were emptied and invertebrates were preserved in 70% ethanol. All wild bee specimens were identified to species level^64^ and validated by experts Mr. Jens D’Haeseleer and Mr. Win Vertommen (Natuurpunt Studie-Mechelen) (Identification of the *Bombus terrestris*-group is difficult, because many Bombus species are cryptic and morphological identification may be impossible between the four different species: *Bombus terrestris, Bombus magnus, Bombus lucorum and Bombus cryptarum*^65^). Wild bee traits were categorised and identified^64,66,67^. Hoverflies were identified to species level^68^ and identification was confirmed by Mr. Ward Tamsyn (Natuurpunt Studie-Mechelen) and Mr. Guy Van de Weyer. Honeybees (*Apis mellifera*) were excluded as they are domesticated bees depending on manmade hives.

### Statistical analysis

Community diversity measures for wild bees were quantified for each roof (data was pooled across the season for each roof), including species richness, abundance, Shannon–Wiener's index (H′), inverse Simpson's diversity index (D), and Pielou evenness (E). To determine whether the respective measures were significantly different between green roofs, Poisson generalized linear mixed models (GLMM) were applied, as Poisson distribution is typically used for count data^69^. Green roof characteristics (vegetation type (categorical), age (continuous), height (continuous), surface area (continuous), percentage of grassland in the surrounding area (continuous)) were used as the fixed factors, roof as the random factor, and each diversity measure (richness, abundance, H′, D, E) as an independent variable. A penalized quasi-likelihood approach was used as a lognormal distribution best fits all responses. Community weighted mean (CWM) trait values for each individual roof were calculated for body size, social behaviour, flower visiting and nesting of wild bee species. The difference in CWM (body size, social behaviour, flower visiting and nesting) values between Sedum roofs and Sedum/herbs/grasses roofs were checked performing sample paired t tests. All data were analysed using R version 3.6.3^70^, and the packages: “vegan”^71^, “matrixStats”^72^, “lme4”^73^ “dplyr”^74^ and “MASS”^75^.

## Results

### Wild bees

In total we collected 597 individuals belonging to 40 different species (Table 2). The average number of species per roof was 8 (x̄ = 7.6, sd = 4.9); roofs housed between two and eighteen species. The number of wild bee individuals sampled per roof varied from three to 168 (x̄ = 29.85, sd = 36.8). The most abundant species were *Lasioglossum laticeps*, *Bombus terrestris*-group and *Hylaeus hyalinatus* (Table 2). Some species such as *Lasioglossum morio* were found on all roofs except four (Onyx, Ell, Eco 2 and Dis). In contrast, twenty species were only found on one roof such as *Andrena cineraria* or *Lasioglossum semiculens* (See Appendix Table A1 in supplementary information).

**Table 2:** Overview of wild bee species found on the green roofs (*B. terrestris-group: *Bombus terrestris* (Linnaeus, 1758), *Bombus lucorum* (Linnaeus, 1761), *Bombus magnus* (Vogt, 1911) and *Bombus cryptarum* (Fabricius, 1755). Abundance: number of individuals caught during the whole sapling period)).

Family	*Genus*	Species	Abundance
Andrenidae	*Andrena*	*barbilabris* (Kirby, 1802)	1
Andrenidae	*Andrena*	*cineraria* (Linnaeus, 1758)	1
Andrenidae	*Andrena*	*dorsata* (Kirby, 1802)	1
Andrenidae	*Andrena*	*minutula* (Kirby, 1802)	2
Andrenidae	*Andrena*	*nitida* (Müller, 1776)	3
Andrenidae	*Panurgus*	*calcaratus* (Kirby, 1802)	1
Apidae	*Anthophora*	*quadrimaculata* (Panzer, 1798)	1
Apidae	*Bombus*	*hortorum* (Linnaeus, 1761)	2
Apidae	*Bombus*	*lapidarius* (Linnaeus, 1758)	27
Apidae	*Bombus*	*pascuorum* (Scopoli, 1763)	55
Apidae	*Bombus*	terrestris-group*	102
Apidae	*Nomada*	*fabriciana* (Linnaeus, 1767)	2
Apidae	*Bombus*	*vestalis* (Geoffroy, 1785)	2
Colletidae	*Colletes*	*daviesanus* (Smith, 1846)	1
Colletidae	*Hylaeus*	*communis* (Nylander, 1852)	3
Colletidae	*Hylaeus*	*hyalinatus* (Smith, 1842)	85
Colletidae	*Hylaeus*	*pictipes* (Nylander, 1852)	5
Halictidae	*Halictus*	*rubicundus* (Christ, 1791)	2
Halictidae	*Halictus*	*scabiosae* (Rossi, 1790)	1
Halictidae	*Lasioglossum*	*fulvicorne* (Kirby, 1802)	5
Halictidae	*Lasioglossum*	*laticeps* (Schenck, 1870)	103
Halictidae	*Lasioglossum*	*leucopus* (Kirby, 1802)	1
Halictidae	*Lasioglossum*	*leucozonium* (Schranck, 1781)	2
Halictidae	*Lasioglossum*	*lucidulum* (Schenck, 1861)	1
Halictidae	*Lasioglossum*	*minutissimum* (Kirby, 1802)	1
Halictidae	*Lasioglossum*	*morio* (Fabricius, 1793)	84
Halictidae	*Lasioglossum*	*nitidulum* (Fabricius, 1804)	52
Halictidae	*Lasioglossum*	*pauxillum* (Schenck, 1853)	1
Halictidae	*Lasioglossum*	*semilucens* (Alfken, 1914)	1
Halictidae	*Lasioglossum*	*sexstrigatum* (Schenck, 1870)	2
Megachilidae	*Anthidium*	*manicatum* (Linnaeus, 1758)	4
Megachilidae	*Chelostoma*	*rapunculi* (Lepeletier, 1841)	2
Megachilidae	*Coelioxys*	*rufescens* (Lepeletier & Serville, 1825)	1
Megachilidae	*Megachile*	*centuncularis* (Linnaeus, 1758)	1
Megachilidae	*Megachile*	*ericetorum* (Lepeletier, 1841)	1
Megachilidae	*Megachile*	*rotundata* (Fabricius, 1787)	24
Megachilidae	*Megachile*	*willughbiella* (Kirby, 1802)	1
Megachilidae	*Osmia*	*bicornis* (Linnaeus, 1758)	5
Megachilidae	*Osmia*	*caerulescens* (Linnaeus, 1758)	1
Melittidae	*Dasypoda*	*hirtipes* (Fabricius, 1793)	7

**Figure 1:**
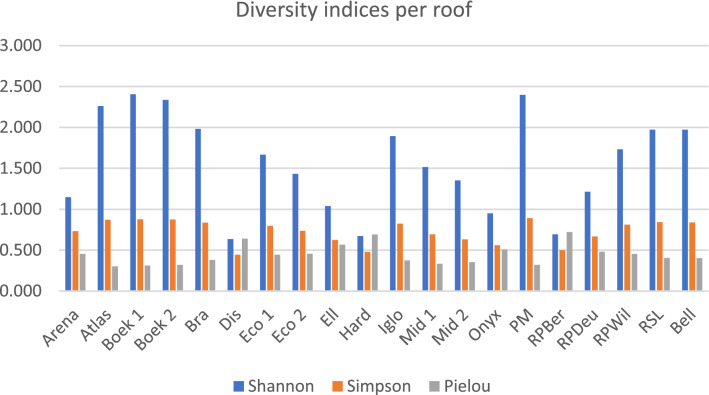
Diversity indices two years combined for each green roof (Shannon–Wiener diversity index: blue bars; Simpson diversity index (inverse): orange bars; Pielou evenness: grey bars).

Most of the individuals (521) were caught with the use of pan traps; only a relatively small number (76 individuals) were caught as by-catch from the pitfall traps. The yellow pan traps attracted the highest numbers (303), while the red pan traps attracted the least individuals, only six (See appendix figure A2 in supplementary information) (Fig. 1).

### Wild bee diversity, species richness and abundance

GLMM results show no significant differences in abundance, species richness and any of the diversity indices between roofs made up of only *Sedum* species and roofs with a combined vegetation cover of *Sedum*, herbs and grasses (Table 3). No significant differences were found when analysing the effect of roof characteristics such as the height, age (i.e. time since construction),surface area and proportion of grassland in the surrounding landscape on the species richness and abundance nor when comparing the two sampling years (Table 4).

**Table 3:** GLMM results of the fixed factor for abundance, species richness and the diversity indices (Shannon Wiener: H′, Simpson: S and Pielou's Evenness: E) between green roofs with a vegetation cover of *Sedum* only and green roofs with a with a mixed vegetation cover of *Sedum*, grass and herbs. Table shows the estimate, standard error (std. Error), Z-value and *p*-value.

	Estimate	SE	Z-value	P-value
Abundance	0.042	0.532	0.064	0.842
Species Richness	1.567	2.214	0.664	0.423
H'	0.078	0.274	0.105	0.811
D	0.034	0.063	0.535	0.899
E	0.017	0.046	0.284	0.674

**Table 4:** Fixed effects table for the generalized linear mixed model (GLMM) detected in the green roof samples for abundance (a) and richness (b) of wild bees (and comparing the two sampling years). Table shows the estimate, standard error (std. Error), Z-value and *p*-value.

	Estimate	SE	Z-value	P-value
*a) Abundance*				
Age	-0.026	0.068	-0.382	0.603
Height	-0.045	0.043	-1.051	0.293
Surface area	0.016	0.001	0.538	0.591
Proportion grassland	0.002	0.034	0.065	0.948
Comparing two years	1.34	6.423	0.241	0.786
*b) Richness*				
Age	0.583	0.446	1.307	0.246
Height	-0.023	0.004	-0.525	0.428
Surface area	-0.003	0.004	-0.809	0.418
Proportion grassland	0.001	0.004	0.445	0.656
Comparing two years	0.046	1.456	0.038	0.964

### Community weighted means of wild bee traits

Bee communities are composed of bee species with certain traits, the typical trait value within the communities for social behaviour (CWM;^76^) indicates that the average wild bee communities on the green roofs are social (see appendix Table A4 in supplementary information).The average bee communities on the green roofs prefer ground nesting. Most species found in this study are polylectic (35 species, abundance 98.9%). Only a small minority of five oligolectic species were found (abundance 1.1%; Table A4). The average CWM for body size ranged between 4.00 mm and 9.75 mm (Table A4). Our results show that wild bee body size does not increase nor decrease significantly with an increase in green roof’s height (See appendix figure A3 in supplementary information). Although statistically not significant (p-value = 0.097), we found a positive trend in CWM average body size with an increase in surface area of the green roof (see appendix figure s4 in supplementary information). Comparison of the two types of roofs (Sedum vs Sedum/herbs/grasses) via dependent sample t-tests showed no significant differences in CWM values (social behaviour: *p*(0.979), nesting: *p*(0.796), flower visit: *p*(0.139) and body length: *p* (0.441)).

### Hoverflies

In total we collected 11 hoverfly individuals from eight different species during the entire sampling period (See appendix Table s5 in supplementary information). All individuals were caught with pan traps. This very low number of individuals allows for no further statistical analysis due to the sample size being too small and increasing the margin of error significantly. Hence, we are unable to include the hoverflies in our further hypothesis testing.

## Discussion

With biodiversity loss occurring at an unprecedented rate^77^ and urbanisation increasing globally, there is an urgent need to optimize urban areas to support biodiversity increase and its ecosystem services, with pollination being a vital one. In this study we explored how green roof characteristics influence the diversity, abundance and species richness of wild bees on these roofs. Our findings can be used to support future biological landscape planning on roofs to optimise pollinator abundances, species composition and diversity in urban areas.

The studied green roofs hosted 40 wild bee species, which represents around 10% of Belgium’s 403 recorded species^78^ and reflects the typical species richness documented globally for studies on green roofs i.e. ranging between 17 and 90 species^25–29,46^.

Our findings did not support our first hypothesis being green roofs with a large surface area have a higher wild bee species richness, abundance and diversity. Larger roofs did not show a positive or a negative effect on species abundance or richness of wild bees (Table 4). It is known that a large number of small green patches, represented by green roofs here, can accumulate species richness even more than a few large patches with an equal total habitat area^79^. As a result, it is important to urge the public to install green roofs, even those with smaller roofs, as these can be evenly beneficial in terms of wild bee species richness than large green roofs. Our results show a slight positive effect on the average body size of wild bee species with an increasing surface area (see appendix figure s4 in supplementary information), but there remains a huge information gap about how urbanisation influences body size changes in wild bee communities and the mechanisms behind these^80^. Furthermore, the findings of our study indicate that the percentage of grassland in the surrounding area has no significant effect on wild bee species abundance or richness on the sampled green roofs (Table 4). This result aligns with a previous study which found that grassland in the surrounding area did not have an impact on arthropod diversity and richness^81^. Additionally, the majority of the green space surrounding our green roofs consists of turf grass, which has been shown to have no significant effect on abundance or diversity of wild bee species on green roofs before^25^. However, with studies that have yielded varying results, it remains uncertain as to the extent to which the surrounding green space impacts the arthropod communities found on green roofs.

Increasing roof height did not affect wild bee abundance nor species richness. When testing for an effect of green roof height on body size, we again could not find any differences. Probably the height of our roofs is not distinctive enough for the wild bees to distinguish for these metrics. Moreover, small variations in vegetation appear to have an effect on the fauna present^82^. We only used roofs reflecting the two most popular types around the world i.e. roofs covered with *Sedum* species only and roofs covered with a combination of *Sedum*, herbs, and grasses, but could not detect significant differences in abundance, species richness or any of the diversity indices of wild bees (Table 3) between both types. Indecisive whether the vegetation plays a role in it, the bee communities on the green roofs were dominated by social species (29 species; Table A4). While some research shows that social bee species are more abundant in urban areas^83,84^, others show that solitary bees are more common in those areas^85^. Our results further add to the findings that the occurrence of this trait is case-specific regarding methodologies and areas of research used in different studies^86^, moreover, the variation in reaction among different bee species to urbanisation adds to this discrepancy. Green roofs in our study seem to be a suitable habitat for ground nesting species in an urban environment (table A4) contrasting the findings that above-ground nesting wild bee species are typical for urban areas^86,87^. The latter is probably due to the presence of a higher number of potential nesting sites^88^. Ground-nesting bees are probably less frequently found in urban areas due to the limitation of suitable nesting sites, the strong human disturbance and their sensitivity to habitat fragmentation. On top of that bare soil patches typically disappear in urban landscapes. On green roofs these patches are typically more present. This study, however, does not allow us to claim that the bees are effectively nesting on the roofs. The bees sampled on our roof could reflect bees that nest in the surrounding landscape and use the roof top as a foraging habitat patch, or bees that nest in the roof and forage on flowers on the roof (or in the surrounding landscape), or bees opportunistically foraging from our pan traps. A previous study on wild bees in the city of Antwerp^89^ also showed a light preference for ground-nesting. However, these results can be explained by the fact that the bee sampling was performed in gardens, parks and cemeteries, where free (undeveloped) soils were still available. It is likely that these places, together with green roofs, act as sanctuaries for ground-nesting wild bee species in an urban environment. The relatively high abundances of *Lasioglossum morio* and *Lasioglossum laticeps* (Table 2) were rather expected as they are quite prevalent in urban settings, including urban green roofs^28,90,91^. Furthermore, other species, such as *Lasioglossum sextrigatum, Hylaeus hyalinatus, Osmia bicornis, Anthidium manicatum, Anthophora quadrimaculata, Megachile centuncularis, Dasyopoda hirtipes*, sampled in this study are also positively associated with urban areas in our region^92^.

*Lasioglossum* species are typically regarded as less efficient pollinators compared to honeybees and bumblebees^93^, due to their smaller body size^94,95^, carrying capacity to transfer pollen grains to stigmas^96^, and their slower movement between flowers. However, due to their large numbers they are still considered as effective pollinators^93,94,97^. Until now *Lasioglossum morio* is known as the only wild bee species that can spend its whole life cycle on green roofs^98^. Interestingly, genera such as *Lasioglossum* appear to be more resilient than other genera, such as *Andrena*, to land-use change^99^, however, other findings show negative effects on *Lasioglossum* species abundance with greater urbanisation^100^. These varying effects of urbanisation can also be seen in the abundance of bumblebees^101,102^. Although we did not study green roofs in less urbanized environments, our findings suggest that *Lasioglossum* and *Bombus* abundances are at least not negatively impacted by urbanisation (Table 2).

Urbanisation in Europe is causing the decline of specialized species^103^. In general bee communities on our green roofs are made of polylectic species and less of oligolectic species (see appendix table A4 and table A2 in supplementary information), as a broader diet is likely best for facilitating species expansion in urban areas^25,104–106^. The specific diet of oligolectic species is rather difficult to maintain in urban settings due to the lack of sufficient plant species to collect pollen. Furthermore, the retrieval of only a few cleptoparasitic species (*Bombus vestalis*, *Ceolioxys rufescens* and *Nomada fabriciana*) corresponds to the findings of Braaker et al.^107^ and Passaseo et al.^108^. Our findings thus indicate that green roofs primarily harbour generalist species rather than specialist species with a higher pollinator effectiveness.

Although our main study objectives focus on wild bees, we share some brief findings on our hoverfly samples. Only 11 hoverfly individuals belonging to eight different species were discovered (See appendix table s5 in supplementary information). Hoverflies occur only in very low diversities and abundances in urban environments^56,109^, including on green roofs^108,110,111^. The lack of sufficient plants, aphids, and decomposing vegetal debris in urban contexts possibly creates a scarcity of larval food supplies. The homogeneous landscape also provides less egg laying sites, making it difficult for species to complete their whole life cycle^56^. Four out of the eight species found (*Episyrphus balteatus*, *Melanostoma mellinum, Scaeva pyrastri* and *Sphaorphoria scripta)* are in fact strong migratory species^112,113^ with less difficulties conquering the habitat isolation of green roofs. Moreover, *E. balteatus, S. pyrastri and Merodon equestris* are highly anthropophilic species and *Helophilus pendulus, S. scripta* and *M. mellinum* are species regularly found in urban or suburban habitats^67,112^. Furthermore, *S. scripta* is known to be one of the few hoverfly species capable of spending their whole life cycle on green roofs^98^. Although it is challenging to draw any conclusions from our results due to the extremely small sample size, our findings indicate that only few hoverflies use green roofs as a suitable habitat. It is incomprehensible that so little research has been done on hoverflies on green roofs or in urban settings in general, when you know that more than 70% of animal-pollinated wildflower species are visited by hoverflies. Future research should in our opinion, move away from concentrating just on honeybees and bumblebees, and instead involve this significant pollinator group more.

Our sampling methodology might have caused some bias in hoverfly and bee observations. Some species, such as *Bombus*, are less commonly caught in pan traps^114^. The integration of active netting together with pan trapping is often suggested as a possible solution for a more exhaustive sampling method of bee and hoverfly populations. However, active netting over multiple sampling sites is extremely labour-intensive for only a single person to sample in the same time frame to ensure for instance similar weather conditions. There is also a large risk in creating additional biases by the sampler and sampling moment if more samplers or different times are used.

As discussed above, green roofs vary depending on their surrounding environment, vegetation cover, height, age, and a variety of other factors. As a result, and due to the large heterogeneity in urban areas, it remains difficult to identify explicitly the true drivers behind our findings and to compare one on one different green roof studies. As mentioned before, the effects of urbanisation on pollinator communities are probably case-specific and differ between regions and climates. Future studies should seek to minimize variability in sampling techniques, study periods, and other methodological differences that may underlie inconsistent results and conclusions. Overall, our results indicate that green roofs in Belgium can be a habitat for a variety of wild bee species in an urban environment. However, when considering hoverflies, green roofs like other urban areas appear to be a less sufficient habitat.

## Supplementary Information

Supplementary Information.

## Data Availability

The datasets generated during and/or analysed during the current study are available from the corresponding author on reasonable request.

## References

[1] Seto KC, Güneralp B, Hutyra LR (2012). Global forecasts of urban expansion to 2030 and direct impacts on biodiversity and carbon pools. Proc. Natl. Acad. Sci. USA.

[2] Faeth SH, Bang C, Saari S (2011). Urban biodiversity: Patterns and mechanisms. Ann. N. Y. Acad. Sci..

[3] Elmqvist T, Zipperer W, Güneralp B, Seta K, Solecki WD, Griffith CA (2016). Urbanisation, habitat loss, biodiversity decline: Solution pathways to break the cycle. Routledge Handbook of Urbanisation and Global Environmental Change.

[4] Dirzo R (2014). Defaunation in the Anthropocene. Science.

[5] Hallmann CA (2017). More than 75 percent decline over 27 years in total flying insect biomass in protected areas. PLoS One.

[6] Wagner D, Grames EM, Forister ML, Berenbaum MR, Stopak D (2021). Insect decline in the Anthropocene: Death by a thousand cuts. Biological sciences.

[7] Goulson D, Nicholls E, Botias C, Rotheray EL (2015). Bee declines driven by combined stress from parasites, pesticides, and lack of flowers. Science.

[8] Ollerton, J. (2021) *Pollinators & pollination: nature and society*. Pelagic publishing.

[9] IPBES (2016). *The assessment report of the Intergovernmental Science-Policy Platform on Biodiversity and Ecosystem Services on pollinators, pollination and food production.*potts, S.G., Imperatriz-Fonseca, V.L and Ngo, H.T. (eds). Secretariat of the Intergovernmental Science-Policy Platform on Biodiversity and Ecosystem Services, Bonn, Germany. 552 pages.

[10] Mallinger RE, Gratton C (2015). Species richness of wild bees, but not the use of managed honeybees, increases fruit set of a pollinator dependent crop. J. Appl. Ecol..

[11] Kremen C, Williams NM, Thorp RW (2002). Crop pollination from native bees at risk from agricultural intensification. Proc. Natl. Acad. Sci. U.S.A..

[12] Winfree R, Fox JW, Williams NM, Reilly JR, Cariveau DP (2015). Abundance of common species, not species richness, drives delivery of a real-world ecosystem service. Ecol. Lett..

[13] Soroye P, Newbold T, Kerr J (2020). Climate change contributes to widespread declines among bumble bees across continents. Science.

[14] Matteson KC, Ascher JS, Langellotto GA (2008). Bee richness and abundance in New York City urban gardens. Ann. Entomol. Soc. Am..

[15] Carré G (2009). Landscape context and habitat type as drivers of bee diversity in European annual crops. Agr. Ecosyst. Environ..

[16] Goulson D, Lye GC, Darvill B (2008). Decline and conservation of bumble bees. Ann. Rev. Entomol..

[17] Bates AJ (2011). Changing bee and hoverfly pollinator assemblages along an urban-rural gradient. PLoS One.

[18] Deguines N, Julliard R, De Flores M, Fontaine C (2016). Functional homogenization of flower visitor communities with urbanisation. Ecol. Evol..

[19] Larsson M (2005). Higher pollinator effectiveness by specialist than generalist flower-visitors of unspecialized Knautia arvensis (Dipsacaceae). Oecologia.

[20] Pataki DE (2011). Coupling biogeochemical cycles in urban environments: Ecosystem services, green solutions, and misconceptions. Front. Ecol. Environ..

[21] Mentens J, Raes D, Hermy M (2006). Green roofs as a tool for solving rainwater runoff problems in the urbanized 21st century?. Landscape Urban Plann..

[22] Oberndorfer E (2007). Green roofs as urban ecosystems: Ecological structures, functions and services. Bioscience.

[23] Braaker S, Ghazoul J, Obrist MK, Moretti M (2014). Habitat connectivity shapes urban arthropod communities: The key role of green roofs. Ecology.

[24] Colla SR, Willis E, Packer I (2009). Can green roofs provide habitat for urban bees (Hymenoptera: Apidae)?. Cities and the Environment.

[25] Tonietto R, Fant J, Ascher J, Ellis K, Larkin D (2011). A comparison of bee communities of Chicago green roofs, parks and prairies. Landsc. Urban Plan..

[26] Ksiazek K, Fant J, Skogen K (2012). An asssement of pollen limitation on Chicago green roofs. Landsc. Urban Plan..

[27] MacIvor JS (2015). Building height matters: Nesting activity of bees and wasps on vegetated roofs. Israel J. Ecol. Evol..

[28] Kratschmer S, Kriechbaum M, Pachinger B (2018). Buzzing on top: Linking wild bee diversity, abundance and traits with green roof qualities. Urban Ecosyst..

[29] MacIvor JS, Ruttan R, Salehi B (2014). Exotics on exotics: Pollen analysis of urban bees visiting Sedum on a green roof. Urban Ecosyst..

[30] Matteson KC, Langellotto GA (2010). Determinates of inner city butterfly and bee species richness. Urban Ecosyst..

[31] Geslin B, Gauzens B, Thébault E, Dajoz I (2013). Plant pollinator networks along a gradient of urbanisation. PLoS One.

[32] Baldock, K.C.R, et al. (2015) Where is the UK's pollinator biodiversity? The importance of urban areas for flower-visiting insects. *Proc. R. Soc.* B. 10.1098/rspb.2014.2849 10.1098/rspb.2014.2849 PMC4345454 25673686

[33] Theodorou P (2020). Urban fragmentation leads to lower floral diversity, with knock-on impacts on bee biodiversity. Sci. Rep..

[34] Lowenstein, D.M., Matteson, K.C., Xiao, I., Silva, A.M. and Minor, E.S (2014) Humans, bees, and pollination services in the city: The case of Chicago, IL (USA). *Biodiversity Conservation* 1–18. 10.1007/s10531-014-0752-0

[35] Winfree R, Bartomeus I, Cariveau D (2011). Native pollinators in anthropogenic habitats. Annu. Rev. Ecol. Evol. Syst..

[36] Cariveau DP, Winfree R (2015). Causes of variation in wild bee responses to anthropogenic drivers. Curr. Opin. Insect. Sci..

[37] Baldock KCR (2019). A systems approach reveals urban pollinator hotspots and conservation opportunities. Nat. Ecol. Evol..

[38] Li WC, Yeung KKA (2021). A comprehensive study of green roof performance from environmental perspective. Int. J. Sustain. Built Environ..

[39] Turner M, Baker WL, Peterson CJ, Peet RK (1998). Factors influencing succession: Lessons from large, infrequent natural disturbances. Ecosystems.

[40] Molineux CJ, Connop SP, Gange AC (2014). Manipulating soil microbial communities in extensive green roof substrates. Sci. Total Environ..

[41] Macivor S, Ksiazek K (2015). Invertebrates on green roofs. Ecol. Stud. Anal. Synthes..

[42] Madre F, Vergnes A, Machon N, Clergeau P (2013). A comparison of 3 types of green roof as habitats for arthropods. Ecol. Eng..

[43] Lee LH, Lin JC (2015). Green roof performance towards good habitat for butterflies in the compact city. Int. J. Biol..

[44] Preston FW (1962). The canonical distribution of commonness and rarity: Part I. Ecology.

[45] Orford KA, Murray PJ, Vaughan IP, Memmott J (2016). Modest enhancements to conventional grassland diversity improve the provision of pollination services. J. Appl. Ecol..

[46] Brenneisen S (2005). The Natural Roof (NADA): Research Project Report on the Use of Extensive Green Roofs by Wild Bees.

[47] Jacobs J, Berg M, Beenaerts N, Artois T (2022). Biodiversity of Collembola on green roofs: A case study of three cities in Belgium. Ecol. Eng..

[48] McKinney, M.L., Sisco, N.D. (2018) Systematic variation in roof spontaneous vegetation: residential “low rise” versus commercial “high rise” buildings*. Urban Nature* **SI,** 73–88.

[49] Rotheray, G.E., & Gilbert, S.F. (2011) *The natural history of hoverflies.* Tresaith, UK: Forrest Text

[50] Benvenuti S (2014). Wildflower green roofs for urban landscaping, ecological sustainability and biodiversity. Landsc. Urban Plan..

[51] Schneider F (1948). Beitrag zur Kenntnis der Generationsverhaltnisse und Diapause rauberischer Schwebfliegen (Syrphldae, Dipt.). Mittl. Schweiz Ent Ges.

[52] Rader R, Edwards W, Westcott DA, Cunningham SA, Howlett BG (2011). Pollen transport differs among bees and flies in a human-modified landscape. Divers. Distrib..

[53] Burgio G, Sommaggio D (2007). Syrphids as landscape bioindicators in Italian agroecosystems. Agr. Ecosyst. Environ..

[54] Doyle T (2020). Pollination by hoverflies in the Anthropocene. Proc. R. Soc. B.

[55] Persson AS, Ekroos J, Olssona P, Smith HG (2020). Wild bees and hoverflies respond differently to urbanisation, human population density and urban form. Landsc. Urban Plann..

[56] Verboven H, Uyttenbroeck R, Brys R, Hermy M (2014). Different responses of bees and hoverflies to land use in an urban–rural gradient show the importance of the nature of the rural land use. Landsc. Urban Plan..

[57] Schönrogge K (2006). Host propagation permits extreme local adaptation in a social parasite of ants. Ecol. Lett..

[58] Schweiger O (2007). Functional richness of local hoverfly communities (Diptera, Syrphidae) in response to land use across temperate Europe. Oikos.

[59] KMI: Koninklijk Meteorologisch Instituut (2022) Analyse van het jaar 2020 en 2021. Available from https://www.meteobelgie.be/klimatologie/waarnemingen-en-analyses/jaar-2020/2274-jaa-2020 (2020) https://www.meteobelgie.be/klimatologie/waarnemingen-en-analyses/jaar-2021/2291-analyse-van-het-jaar-2021 (2021). Accessed on 12/05/2022.

[60] Shrestha M (2019). Fluorescent pan traps affect the capture rate of insect orders in different ways. Insects.

[61] Cooper, R., & Whitmore, R.C. (1990) *Arthropod sampling methods in ornithology, Avian Foraging: theory, methodology, and applications.* Studies in Avian Biology 13, Cooper Ornithological Society, California.

[62] Oberprieler SK, Andersen A, Braby MF (2019). Invertebrate by-catch from vertebrate pitfall rraps can be useful for documenting patterns of invertebrate diversity. J. Insect. Conserv..

[63] Skvarla MJ, Larson JL, Dowling APG (2014). Pitfalls and preservatives: A review. J. Entomol. Soc. Ontario.

[64] Michez, D., Rasmont, P., Terzo, M. and Vereecken, N.J. (2019) *Bees of Europe. Hymenoptera of Europe 1*. N.A.P Editions.

[65] Williams, P.H., et al. (2012): Unveiling cryptic species of the bumblebee subgenus Bombus s. str. worldwide with COI barcodes (Hymenoptera: Apidae). *Syste. Biodiversity*. 10.1080/14772000.2012.66457

[66] Falck, S., & Lewington, R (2020) *Bijen veldgids voor Nederland en Vlaanderen.* Tirion.

[67] Koster, A. (2022) De Nederlandse wilde bijen en hun planten. http://www.denederlandsebijen.nl/. Accessed on 21/4/2022.

[68] Speight, M.C.D. & Sarthou, J.P. (2013) *StN keys for the identification of adult European Syrphidae (Diptera) 2013/Clés StN pour la détermination des adultes des Syrphidae Européens (Diptères) 2013.* Syrph the Net, the database of European Syrphidae, Vol. 74, 133pp, Syrph the Net publications, Dublin.

[69] Roback, P., Legler, J. (2021) *Beyond Multiple Linear Regression: Applied Generalized Linear Models and Multilevel Models in R*. Taylor & Francis Group, LLC.

[70] R Core Team (2020) *R: A Language and Environment for Statistical Computing*. R Foundation for Statistical Computing, Vienna, Austria.

[71] Oksanen, J., et al. (2014) Vegan: community ecology package. R Package 280.

[72] Bengtsson, H. (2017). matrixStats: Functions that Apply to Rows and Columns of Matrices (and to Vectors). R Package Version 0.52.2.

[73] Bates, D., Mächler, M., Bolker, B., & Walker, S. (2015) Fitting linear mixed-effects models using lme4. *J. Stat. Softw.* **67(1),** 1–48. 10.18637/jss.v067.i01.

[74] Wickham, H., François, R., Henry, L. and Müller, K. (2022). dplyr: A Grammar of Data Manipulation. https://dplyr.tidyverse.org, https://github.com/tidyverse/dplyr.

[75] Venables, W.N., & Ripley, B.D. (2002) *Modern Applied Statistics with S*, 4th ed. Springer, New York. ISBN 0–387–95457–0. https://www.stats.ox.ac.uk/pub/MASS4/.

[76] Ricotta C, Moretti M (2011). CWM and Rao's quadratic diversity: A unified framework for functional ecology. Oecologia.

[77] Leclère D (2020). Bending the curve of terrestrial biodiversity needs an integrated strategy. Nature.

[78] Drossart, M., et al. (2019) *Belgian red list of Bees*. Belgian Science Policy (BRAIN-be - (Belgian Research Action through Interdisciplinary Networks). Mons: Presse universitaire de l’Université de Mons.

[79] Fahrig L (2020). Why do several small patches hold more species than few large patches?. Glob. Ecol. Biogeogr..

[80] Ayers AC, Rehan SM (2021). Supporting bees in cities: how bees are influenced by local and landscape features. Insects.

[81] Domínguez, M. V. S., González, E., Fabián, D., Salvo, A. & Fenoglio, M. S. Arthropod diversity and ecological processes on green roofs in a semi-rural area of Argentina: Similarity to neighbor ground habitats and landscape effects. *Landscape and Urban Planning* **199**, (2020).

[82] Castagneyrol B, Jactel H (2012). Unravelling plant- animal diversity relationships: A meta-regression analysis. Ecology.

[83] Harrison T, Gibbs J, Winfree R (2018). Phylogenetic homogenization of bee communities across ecoregions. Glob. Ecol. Biogeogr..

[84] Wenzel A, Grass I, Belavadi VV, Tscharntke T (2020). How urbanisation is driving pollinator diversity and pollination, a systematic review. Biol. Conserv..

[85] Martins KT, Gonzalez A, Lechowicz MJ (2017). Patterns of pollinator turnover and increasing diversity associated with urban habitats. Urban Ecosyst..

[86] Bucholz S, Egerer M (2020). Functional ecology of wild bees in cities: Towards a better understanding of trait-urbanisation relationships. Biodiver. Conserv..

[87] Hernandez JL, Frankie GW, Thorp RW (2009). Ecology of urban bees : A review of current knowledge and directions for future study. Cities Environ..

[88] Cane JH, Johnson EA, Klemens MW (2005). Bees, pollination, and the challenges of sprawl. Nature in fragments: The legacy of sprawl.

[89] Koch K (2014). Wilde bijensoorten in een stedelijke omgeving: Stad Antwerpen. Antenna.

[90] Soper J, Beggs J (2013). Assessing the impact of an introduced bee, Anthidium manicatum, on pollinator communities in New Zealand. NZ J. Bot..

[91] Bennet, D.G., Kelly, D., & Clemens, J. (2018). Food plants and foraging distances for the native bee *Lasioglossum sordidum* in Christchurch Botanic Gardens. *New Zealand J. Ecol.* **42(1),** 40–47. 10.20417/nzjecol.42.1

[92] Vanormelingen, P., Remer, M., & D’Haeseleer, J. (2021) Wilde bijen en bebouwing: meer verliezers dan winnaars? *Themanummer bijen in de stad en dorp, Hymenovaria*, maart 2021.

[93] Rader R (2009). Alternative pollinator taxa are equally efficient but not as effective as the honey-bee in a mass flowering crop. J. Appl. Ecol..

[94] Garantonakis N (2016). Comparing the pollination services of honey bees and wild bees in a watermelon field. Sci. Hortic..

[95] Foldesi R, Howlett BG, Grass I, Batary P (2021). Larger pollinators deposit more pollen on stigmas across multiple plant species – A meta-analysis. J. Appl. Ecol..

[96] Howlett, et al. (2011). Can insect body pollen counts be used to estimate pollen deposition on pak choi stigmas? *New Zealand Plant Protection* **64,** 25–31. 10.30843/nzpp.2011.64.5951

[97] Nelson W, Barry Donovan LE, Howlett B (2022). Lasioglossum bees – the forgotten pollinators. J. Apic. Res..

[98] Passaseo A, Pétremand G, Rochefort S, Castella E (2020). Pollinators emerging from extensive green roofs: Wild bees (Hymenoptera: Antophila) and hoverflies (Diptera: Syrphidae) in Geneva (Switzerland). Urban Ecosyst..

[99] Odanaka KA, Rehan SM (2019). Impact indicators: Effects of land use management on functional trait and phylogenetic diversity of wild bees. Agric. Ecosyst. Environ..

[100] Wilson CJ, Jamieson MA (2019). The effects of urbanisation on bee communities depends on floral resource availability and bee functional traits. PLoS ONE.

[101] Osborne JL (2007). Quantifying and comparing bumblebee nest densities in gardens and countryside habitats. J. Appl. Ecol..

[102] Glaum P, Simao MC, Vaidya C, Fitch G, Lulinao B (2017). Big city Bombus: Using natural history and land-use history to find significant environmental drivers in bumble-bee declines in urban development. R Soc Open Sci..

[103] Rasmont P (2015). Climatic risk and distribution atlas of European bumblebees. Biorisk.

[104] Roger N (2017). Impact of pollen resources drift on common bumblebees in NW Europe. Glob. Change Biol..

[105] Frankie GW (2005). Ecological patterns of bees and their host ornamental flowers in two northern California cities. J. Kansas Entomol. Soc..

[106] Lerman SB, Milam J (2016). Bee fauna and floral abundance within lawn-dominated suburban yards in Springfield, MA. Ann. Entomol. Soc. Am..

[107] Braaker S, Obrist MK, Ghazoul J, Moretti M (2017). Habitat connectivity and local conditions shape taxonomic and functional diversity of arthropods on green roofs. J. Anim. Ecol..

[108] Passaseo, A., Rochefort, S., Pétremand, G., & Castella, E. (2021) Pollinators on green roofs: Diversity and trait analysis of wild bees (Hymenoptera: Anthophila) and Hoverflies (Diptera: Syrphidae) in an urban area (Geneva, Switzerland). *Cities and the Environment (CATE)*10.15365/cate.2021.140201

[109] Hennig E, Ghazoul J (2012). Pollinating animals in the urban environment. Urban Ecosyst..

[110] Mecke R. (1996) *Die fauna begrünter dächer: Ökologische untersuchung verschiedener dachflächer im Hamburger stadtgebiet.* University of Hamburg, Diploma dissertation.

[111] Bevk D (2021). The diversity of pollinators on green roofs. Acta Entomol. Slovenica.

[112] Speight, M.C.D. (2011) *Species accounts of European Syrphidae (Diptera), Glasgow 2011*. Syrph the Net, the database of European Syrphidae, vol. 65, 285 pp., Syrph the Net publications, Dublin.

[113] Wotton KR (2019). Mass seasonal migrations of hoverflies provide extensive pollination and crop protection services. Curr. Biol..

[114] Boyer KJ, Fragoso FP, Mabin MED, Brunet J (2020). Netting and pan traps fail to identify the pollinator guild of an agricultural crop. Nat. Res. Sci. Rep..

