# Supplementary material for: Green roofs and pollinators, useful green spots for some wild bee species (Hymenoptera: Anthophila), but not so much for hoverflies (Diptera: Syrphidae)
Source: Sci Rep. 2023 Jan 26;13:1449. doi: 10.1038/s41598-023-28698-7 (PMC9879974; doi:10.1038/s41598-023-28698-7)
Supplement: Supplementary file 1 — Supplementary Information. [file 41598_2023_28698_MOESM1_ESM.docx]

Green roofs and pollinators, useful green spots for some wild bee species (Hymenoptera: Anthophila), but not so much for hoverflies (Diptera: Syrphidae). Jeffrey Jacobs, Prof. Dr. Natalie Beenaerts and Prof Dr. Tom Artois

Appendix


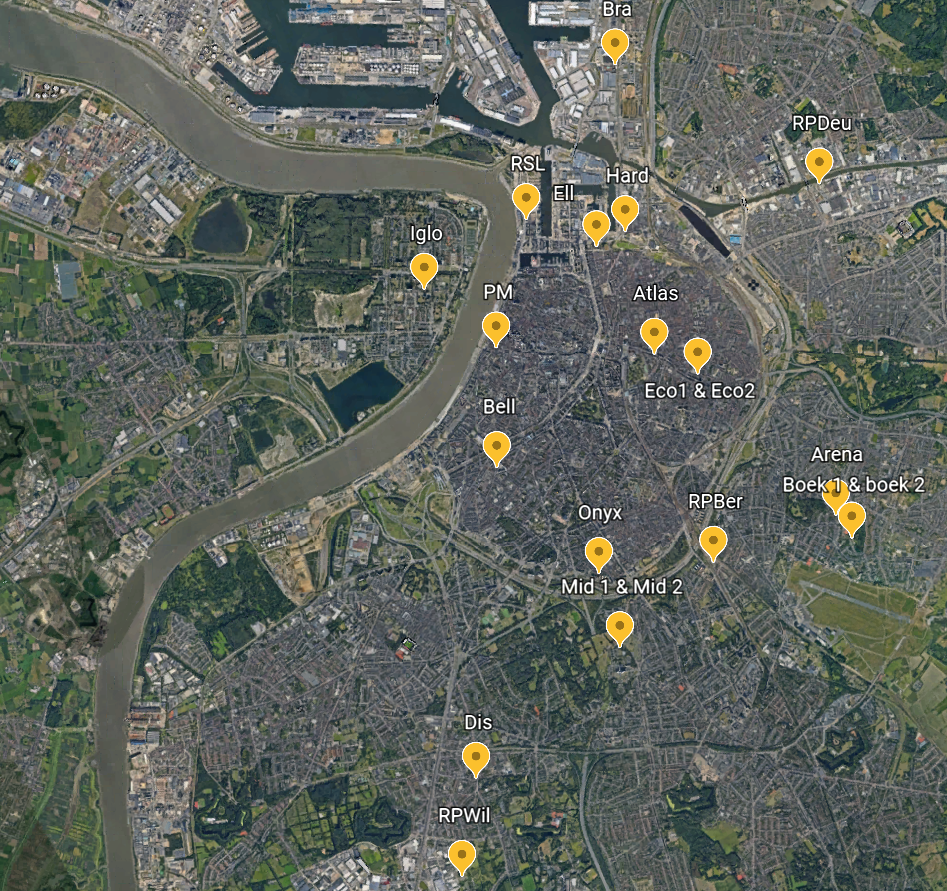


Figure A1: Map of the city of Antwerp and location of the roofs we investigated. (image: Maps data ©Google, 2022, Antwerp, Belgium. Available at https://www.google.be/maps)

Figure A2: Number of wild bee individuals sampled per pan trap-type (colour) during the whole sampling period.

Table A1: Species found on each roof, (number of individuals per roof).

Figure A3: Effect of green roof’s height on the average (CWM) body size of wild bee species.

Figure A4: Effect of green roof’s surface area on the average (CWM) body size of wild bee species.

Table A2: overview of the species found on the green roofs with their average body length, social behaviour, flower visit and nesting.

| **Genus** | **Species** | **Average body length** | **Social behaviour** | **Flower visit** | **Nesting** |
| --- | --- | --- | --- | --- | --- |
| *Andrena* | *barbilabris* (Kirby, 1802) | 7.5mm | Solitary | oligolectic | Ground-nesting |
| *Andrena* | *cineraria* (Linnaeus, 1758) | 10mm | Solitary | Polylectic | Ground-nesting |
| *Andrena* | *dorsata* (Kirby, 1802) | 7mm | Solitary | Polylectic | Ground-nesting |
| *Andrena* | *minutula* (Kirby, 1802) | 5mm | Solitary | Polylectic | Ground-nesting |
| *Andrena* | *nitida* (Müller, 1776) | 10mm | Solitary | Polylectic | Ground-nesting |
| *Anthidium* | *manicatum* (Linnaeus, 1758) | 10.5mm | Solitary | Polylectic | Ground-nesting |
| *Anthophora* | *quadrimaculata* (Panzer, 1798) | 8mm | Solitary | Polylectic | Ground-nesting |
| *Bombus* | *hortorum* (Linnaeus, 1761) | 14mm | Social | Polylectic | Above and Ground-nesting |
| *Bombus* | *lapidarius* (Linnaeus, 1758) | 12mm | Social | Polylectic | Above-ground |
| *Bombus* | *pascuorum* (Scopoli, 1763) | 11mm | Social | Polylectic | Above and Ground-nesting |
| *Bombus* | *terrestris-groep* | 13mm | Social | Polylectic | Ground-nesting |
| *Bombus* | *vestalis* (Geoffroy, 1785) | 16mm | Social | Polylectic | Above and Ground-nesting |
| *Chelostoma* | *rapunculi* (Lepeletier, 1841) | 7mm | Solitary | Oligolectic | Above-ground |
| *Coelioxys* | *rufescens* (Lepeletier & Serville, 1825) | 7mm | Solitary | Polylectic | Above-ground |
| *Colletes* | *daviesanus* (Smith, 1846) | 6.5mm | Solitary | Oligolectic | Above-ground |
| *Dasypoda* | *hirtipes* (Fabricius, 1793) | 10mm | Solitary | Polylectic | Ground-nesting |
| *Halictus* | *rubicundus* (Christ, 1791) | 7.5mm | Primitive Social | Polylectic | Ground-nesting |
| *Halictus* | *scabiosae* (Rossi, 1790) | 9mm | Primitive Social | Polylectic | Ground-nesting |
| *Hylaeus* | *communis* (Nylander, 1852) | 5mm | Solitary | Polylectic | Above-ground |
| *Hylaeus* | *hyalinatus* (Smith, 1842) | 4mm | Solitary | Polylectic | Above and Ground-nesting |
| *Hylaeus* | *pictipes* (Nylander, 1852) | 3.5mm | Solitary | Polylectic | Above-ground |
| *Lasioglossum* | *fulvicorne* (Kirby, 1802) | 5mm | Solitary | Polylectic | Ground-nesting |
| *Lasioglossum* | *laticeps* (Schenck, 1870) | 5mm | Primitive Social | Polylectic | Ground-nesting |
| *Lasioglossum* | *leucopus* (Kirby, 1802) | 4mm | Solitary | Polylectic | Ground-nesting |
| *Lasioglossum* | *leucozonium* (Schranck, 1781) | 5.5mm | Solitary | Polylectic | Ground-nesting |
| *Lasioglossum* | *lucidulum* (Schenck, 1861) | 3.5mm | Solitary | Polylectic | Ground-nesting |
| *Lasioglossum* | *minutissimum* (Kirby, 1802) | 3.5mm | Solitary | Polylectic | Ground-nesting |
| *Lasioglossum* | *morio* (Fabricius, 1793) | 4mm | Social | Polylectic | Ground-nesting |
| *Lasioglossum* | *nitidulum* (Fabricius, 1804) | 5mm | Solitary | Polylectic | Ground-nesting |
| *Lasioglossum* | *pauxillum* (Schenck, 1853) | 4mm | Social | Polylectic | Ground-nesting |
| *Lasioglossum* | *semilucens* (Alfken, 1914) | 4mm | Solitary | Polylectic | Ground-nesting |
| *Lasioglossum* | *sexstrigatum* (Schenck, 1870) | 5mm | Solitary | Polylectic | Ground-nesting |
| *Megachile* | *centuncularis* (Linnaeus, 1758) | 7.5mm | Solitary | Polylectic | Above and Ground-nesting |
| *Megachile* | *ericetorum* (Lepeletier, 1841) | 9.5mm | Solitary | Oligolectic | Above and Ground-nesting |
| *Megachile* | *rotundata* (Fabricius, 1787) | 6mm | Solitary | Polylectic | Above and Ground-nesting |
| *Megachile* | *willughbiella* (Kirby, 1802) | 8.5mm | Solitary | Polylectic | Above and Ground-nesting |
| *Nomada* | *fabriciana* (Linnaeus, 1767) | 6.5mm | Solitary | Polylectic | Ground-nesting |
| *Osmia* | *bicornis* (Linnaeus, 1758) | 8mm | Solitary | Polylectic | Above-ground |
| *Osmia* | *caerulescens* (Linnaeus, 1758) | 6.5mm | Solitary | Polylectic | Above-ground |
| *Panurgus* | *calcaratus* (Kirby, 1802) | 7.5mm | Solitary | Oligolectic | Ground-nesting |

Table A3: Overview of the green roofs with their vegetation cover (in bold the species that was dominant on each green roof (% coverage of the total green roof area)). Species that made up less than 1% of the total roof area were excluded from this table (Average was taken from the two survey moments in June 2020 and June 2021).

| **Roof** | **Flora** |
| --- | --- |
| Arena | ***Sedum hispanicum*** (Linnaeus, 1755) *(40%), Sedum spurium* (Bieb, 1808)*, Sedum album* (Linnaeus 1753)*,* Bryophyta |
| Atlas | ***Sedum spurium*** *(25%),* ***Vulpia myuros*** (Linnaeus, 1753) *(25%), Sedum album, Sedum hispanicum, Sedum telephium* (Ohba, 1977)*, Sedum sexangulare* (Linnaeus, 1753)*, Cirsium arvense* (Scop, 1772)*, Equisetum arvense* (Linnaeus, 1753)*, Calamagrostis epigejos* (Roth, 1788)*, Teucrium chamaedrys* (Linnaeus, 1753)*, Verbascum densiflorum* (Bertol)*, Thymus pulegioides* (Linnaeus, 1753)*, Iris spp.* (Tournefort ex L.)*, Aira praecox* (Linnaeus, 1753) |
| Bell | ***Sedum album*** *(93%), Sedum spurium, Sedum sexangulare, Senecio inaequidens* (DC, 1838)*, Geranium robertianum* (Linnaeus) |
| Boek 1 | ***Sedum sexangulare*** *(40%), Sedum album, Sedum hispanicum, Sedum spurium, Sedum katschaticum* (Fisch)*, Sedum rupestre* (PV Health)*, Dianthus carthusianorum* (Linnaeus)*, Trifolium arvense* (Linnaeus)*, Petrorhagia prolifera* (PW Ball & Heywood)*, Trisetum flavescens* (P Beauv) |
| Boek 2 | ***Sedum hispanicum*** *(25%),* ***Sedum spurium*** *(25%),* ***Sedum sexangulare*** *(25%), Sedum album, Sedum katschaticum, Sedum rupestre, Petrorhagia prolifera, Trifolium arvense,* Bryophyta |
| Bra | ***Sedum hispanicum*** *(40%) Sedum album, Sedum rupestre, Sedum spurium, Trifolium arvense, Senecio inaequidens, Vulpia myuros,* Bryophyta |
| Dis | ***Sedum album*** *(60%), Sedum Hispanicum, Sedum spurium, Sedum sexangulare, Senecio inaequidens, Sonchus asper* (Hill, 1769)*, Trifolium arvense* |
| Eco 1 | **Sedum hispanicum** (85%), Sedum album, Sedum sexangulare |
| Eco 2 | ***Carex spp****. (50%), Sedum hispanicum, Stipa tenuifolia* (Barkworth) |
| Ell | ***Sedum album*** *(25%),* ***Sedum spurium*** *(25%),* ***Allium schoenoprasum****(25%), Sedum sexangulare, Sedum hybridum, Sedum kamtschaticum, Senecio inaequidens, Festuca filiformis* (Pourret)*, Dianthus carthusianorum,* Bryophyta |
| Hard | ***Sedum hispanicum*** *(40%), Sedum album, Senecio inaequidens, Digitaria sanguinalis* (Scopoli)*, Sonchus oleraceus* (Linnaeus, 1753)*, Sedum spurium, Sedum sexangulare,* Bryophyta |
| Iglo | ***Sedum album*** *(85%), Sedum hispanicum, Sedum spurium, Sedum sexangulare, Sedum katschaticum* |
| Mid 1 | ***Sedum album*** *(50%), Sedum hispanicum, Sedum spurium, Sedum sexangulare, Sedum hyrbridum, Sedum katschaticum* |
| Mid 2 | ***Sedum album*** *(80%), Sedum hispanicum, Sedum spurium, Sedum sexangulare, Sedum hyrbridum, Sedum katschaticum, Vulpia myuros* |
| Onyx | ***Sedum Album*** *(95%), Sedum hybridum, Sedum spurium* |
| PM | ***Sedum hispanicum*** *(75%), Sedum album, Sedum spurium, Sedum acre* (Linnaeus, 1753)*, Conyza canadensis* (Linnaeus, 1753)medic*,* Bryophyta |
| RPBer | ***Sedum album*** *(95%), Sedum hispanicum, Sedum spurium, Sedum sexangulare, Sedum katschaticum* |
| RPDeu | ***Sedum album*** *(80%), Sedum hispanicum, Sedum spurium, Sedum sexangulare, Sedum hybridum, Sedum katschaticum* |
| RPWil | ***Sedum spurium*** *(60%)****,*** *Sedum album, Sedum hispanicum, Sedum sexangulare, Sedum hybridum, Sedum rupestre, Medicago lupulina* (Linnaeus, 1753)*, Trifolium arvense,* Bryophyta |
| RSL | ***Sedum album*** *(60%), Sedum hispanicum, Sedum spurium, Sedum sexangulare, Sedum kamtschaticum,* Bryophyta |

Figure A5: floristic richness per green roof, y-axis shows the number of flora species and x-axis each green roof sampled during our study. First eleven roofs are *Sedum* roofs (Arena, Bell, Eco1, Iglo, Mid1, Mid2, Onyx, PM, RPBer, RPDeu, RSL), last nine roofs are *Sedum*/herbs/grasses roofs (Atlas, Boek1, Boek2, Bra, Dis, Eco2, Ell, Hard, RPWil).

Table A4: Community weighted means (CWM) per roof for sociality, nesting type, pollen specialisation and body size (mm) over all species.

| **Roof** | **Sociality** | **Nesting type** | **Pollen specialisation** | **Body size (mm)** |
| --- | --- | --- | --- | --- |
| Arena | Solitary | Ground-nesting | Polylectic | 4.76 |
| Atlas | Social | Ground-nesting | Polylectic | 6.44 |
| Bell | Social | Ground-nesting | Polylectic | 4.91 |
| Boek 1 | Social | Ground-nesting | Polylectic | 7.38 |
| Boek 2 | Social | Ground-nesting | Polylectic | 7.99 |
| Bra | Social | Ground-nesting | Polylectic | 8.00 |
| Dis | Social | Ground-nesting | Polylectic | 8.50 |
| Eco 1 | Social | Ground-nesting | Polylectic | 5.78 |
| Eco 2 | Solitary | Ground-nesting | Polylectic | 6.56 |
| Ell | Social | Ground-nesting | Polylectic | 9.00 |
| Hard | Social | Ground-nesting | Polylectic | 7.50 |
| Iglo | Social | Ground-nesting | Polylectic | 8.48 |
| Mid 1 | Social | Ground-nesting | Polylectic | 7.55 |
| Mid 2 | Social | Ground-nesting | Polylectic | 9.75 |
| Onyx | Social | Ground-nesting | Polylectic | 9.60 |
| PM | Social | Ground-nesting | Polylectic | 7.10 |
| RPBer | Social | Above-ground | Polylectic | 4.00 |
| RPDeu | Social | Ground-nesting | Polylectic | 9.11 |
| RPWil | Social | Above-ground | Polylectic | 7.63 |
| RSL | Social | Ground-nesting | Polylectic | 7.67 |

Table A5: Hoverfly species found on green roofs, with their respective sampling month.

| **Roof** | **Month-year** | **Species** | **Count** |
| --- | --- | --- | --- |
| Atlas | Jun-21 | *Merodon equestris* (Fabricius, 1794) | 1 |
| Atlas | Sep-21 | *Helophilus pendulus* (Linnaeus, 1758) | 1 |
| Atlas | Sep-21 | *Eristalis sepulcharis* (Linnaeus, 1758) | 1 |
| Atlas | Sep-21 | *Orthonevra brevicornis* (Loew, 1843) | 1 |
| Boek 1 | Jun-20 | *Melanostoma mellinum* (Linnaeus, 1758) | 1 |
| Boek 2 | Aug-21 | *Sphaorphoria scripta* (Linnaeus, 1758) | 1 |
| Boek 2 | Aug-21 | *Episyrphus balteatus* (De Geer, 1776) | 1 |
| Bra | Jun-21 | *Sphaorphoria scripta* (Linnaeus, 1758) | 1 |
| Iglo | Jul-20 | *Scaeva pyrastri* (Linnaeus, 1758) | 1 |
| Iglo | Aug-21 | *Episyrphus balteatus* (De Geer, 1776) | 1 |
| PM | Jul-21 | *Sphaorphoria scripta* (Linnaeus, 1758) | 1 |

Table A6 Model outputs for the generalized linear mixed models (GLMM’s) (for table 3 and 4 from the main text). Table shows the estimate, standard error (std. Error), Z-value, *p*-value, random effect variance (Standard deviation) and degrees of freedom (DF).

| ***Table 3*** | **Estimate** | **std. Error** | **Z-value** | **P-value** | **Random effect variance (with SD)** | **DF** |
| --- | --- | --- | --- | --- | --- | --- |
| *Abundance* | 0.042 | 0.532 | 0.064 | 0.842 | 0.931 (SD= 0.965) | 13 |
| *Species Richness* | 1.567 | 2.214 | 0.664 | 0.423 | 0.248 (SD=0.498) | 13 |
| *H'* | 0.078 | 0.274 | 0.105 | 0.811 | 0.384 (SD= 0.497) | 13 |
| *D* | 0.034 | 0.063 | 0.535 | 0.899 | 0.218 (SD= 0.381) | 13 |
| *E* | 0.017 | 0.046 | 0.284 | 0.674 | 0.021 (SD= 0.125) | 13 |

| **Table 4** | **Estimate** | **std. Error** | **Z-value** | **P-value** | **Random effect variance (with SD)** | **DF** |
| --- | --- | --- | --- | --- | --- | --- |
| Abundance |  |  |  |  |  |  |
| Age | -0.026 | 0.068 | -0.382 | 0.603 | 0.931 (SD= 0.965) | 13 |
| Height | -0.045 | 0.043 | -1.051 | 0.293 | 0.931 (SD= 0.965) | 13 |
| Surface area | 0.016 | 0.001 | 0.538 | 0.591 | 0.931 (SD= 0.965) | 13 |
| Proportion grassland | 0.002 | 0.034 | 0.065 | 0.948 | 0.931 (SD= 0.965) | 13 |
| Comparing two years | 1.340 | 6.423 | 0.241 | 0.786 | 1.121 (SD= 1.002) | 32 |
| Richness |  |  |  |  |  |  |
| Age | 0.583 | 0.446 | 1.307 | 0.246 | 0.248 (SD=0.498) | 13 |
| Height | -0.023 | 0.004 | -0.525 | 0.428 | 0.248 (SD=0.498) | 13 |
| Surface area | -0.003 | 0.004 | -0.809 | 0.418 | 0.248 (SD=0.498) | 13 |
| Proportion grassland | 0.001 | 0.004 | 0.445 | 0.656 | 0.248 (SD=0.498) | 13 |
| Comparing two years | 0.046 | 1.456 | 0.038 | 0.964 | 0.354 (SD=0.687) | 32 |


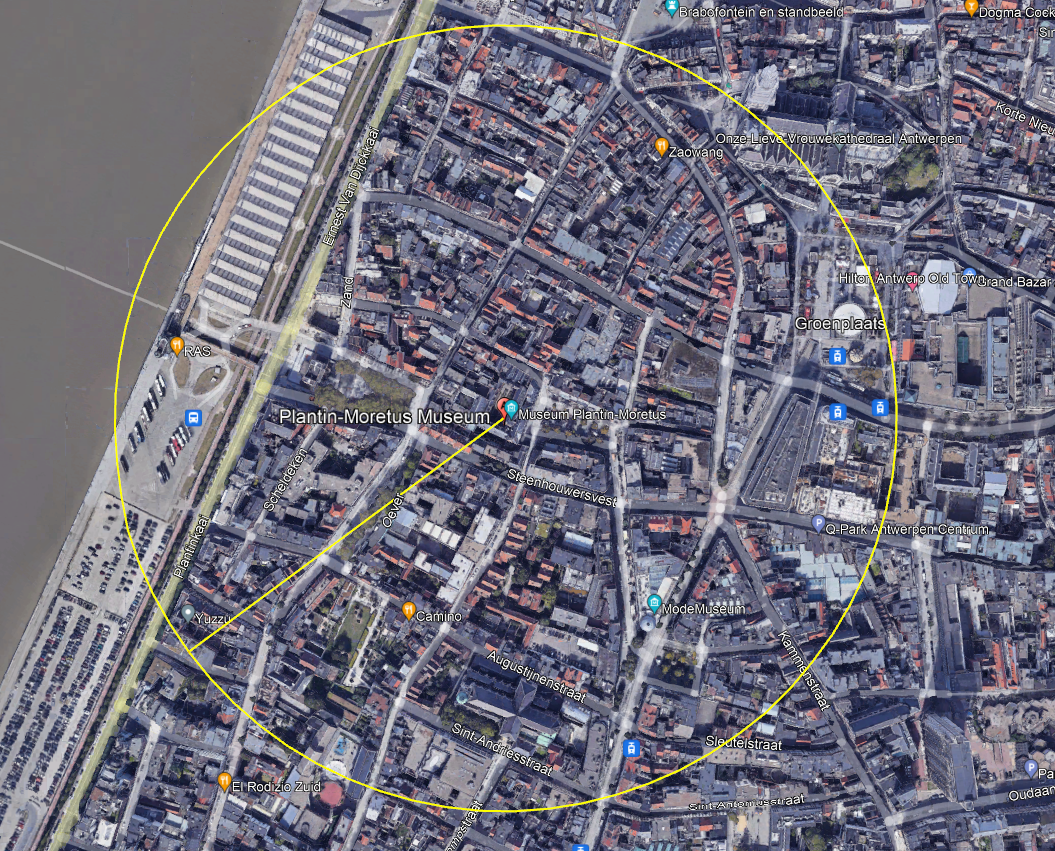


Figure A6: A satellite image in Google Earth Pro (green roof PM) with a radius of 300 meters is shown. The percentage of grassland within this radius was calculated for each individual green roof. (©Google Earth Pro, version 7.3.6.9345, January 6, 2023, Antwerp, Belgium)

Table A7 Overview of the green roofs with their respective percentage of grassland in the surrounding landscape (radius of 300m from the centre of each green roof).

| **Roof** | **Percentage grassland in a 300m radius** |
| --- | --- |
| Arena | 2.3 |
| Atlas | 0.4 |
| Boek 1 | 2.8 |
| Boek 2 | 2.8 |
| Bra | 2.2 |
| Dis | 1.3 |
| Eco 1 | 0.4 |
| Eco 2 | 0.4 |
| Ell | 3.7 |
| Hard | 9.3 |
| Iglo | 14.9 |
| Mid 1 | 23.4 |
| Mid 2 | 23.2 |
| Onyx | 4.4 |
| PM | 2.1 |
| RPBer | 1.7 |
| RPDeu | 1.8 |
| RPWil | 11.8 |
| RSL | 0.5 |
| Bell | 0.7 |
